# Supplementary material for: “If I Was Educated, I Would Call the Ambulance and Give Birth at the Health Facility”—A Qualitative Exploratory Study of Inequities in the Utilization of Maternal, Newborn, and Child Health Services in Northern Ethiopia
Source: Int J Environ Res Public Health. 2022 Sep 15;19(18):11633. doi: 10.3390/ijerph191811633 (PMC9517196; doi:10.3390/ijerph191811633)
Supplement: Supplementary file 1 [file ijerph-19-11633-s001.zip › ijerph-1749257-supplementary.pdf]

Supplementary File S1

Qualitative tools

In-depth Interview (IDI)

Title: Perceptions of inequity in the provision and utilization of maternal, newborn, and child health services in Tigray, Ethiopia. A qualitative explorative study

Good morning/afternoon. My name is Mr. __________________. Thank you for consenting to participate in this study. I am from Mekelle University conducting a study on "Perceptions of inequity in the provision and utilization of maternal, newborn, and child health services in Tigray, Ethiopia. A qualitative explorative study" as part of doctoral studies. The purpose of the study is to explore the perception on equity in maternal፣ newborn and child health services provision and utilization at primary health care facilities. I will also explore why some people in this community do not use health services. To collect information on this topic, you are purposively selected for this study. Thus, we would like you to talk openly and honestly to the best of your knowledge about equity in maternal and child health services utilization. I am interested in all your ideas, comments, and suggestions.

In conducting this study, we are talking to mothers who have live births last year, women development groups, health extension workers, and health workers at different levels who are directly involved in overseeing the maternal and child health services. As a mother who is supposed to utilize the health services, I would like to ask you some questions on the provision and utilization of the overall services concerning the health of mothers and children under five years old. It mainly assesses the equitability of maternal and child health services utilization at health facilities. There are no wrong or right answers. All comments, both positive and negative are welcome. Hence, I would like you to take this seriously to share your opinions and ideas openly and truthfully.

Your participation in this study is voluntary. You may choose to decline to answer questions that you are not comfortable with. There is no direct benefit for your participation in this study.

However, the findings will be essential to improve the equitability of the maternal, newborn and child health services provision and utilization at the primary health care facilities. We encourage you to participate in this study openly and candidly. I assure you that all whatever you talk about will be confidential that will be used for the intended purpose only. It would also share with policy-makers to encourage them to make appropriate decisions that will help the people of these communities.

We will audiotape all your comments and opinions so that we could not miss any of your ideas while trying to take notes. Thus, we will use tape-recorder to pick up each of your suggestions and comments. Any of you who wish to have access to this recording to verify is free to do so. Therefore, before we proceed, we would like to ask you if there are any concern in using the tape recorder. Check for objections, if none, proceed signing the consent form below.

Thank you once again for your cooperation.

Consent of a mother

I understand that the purpose of the study is to collect information regarding the perception of clients on equity in maternal and child health services utilization by the caregivers/mothers who have live births during the last year at the health posts. I have read, or it has been read to me the In-depth interview guide and purpose of the study and understand the explanation of what it is about. I have had the opportunity to ask questions and any questions that I have asked have been answered to my satisfaction. I have consented voluntarily to participate in this study and understand that I have the right to withdraw at any time without in any way affecting my social life or medical care.

Thank you for your participation.

Signature of informant _________________________ Date ____________________

Signature of interviewer ___________________________ Date ____________________

In-depth interview guide for mothers who have ever visited the health facilities for any maternal and child health-related services.
Study Title: "Experience and Perception of clients on Equity in maternal, neonatal and child health services utilization at the primary healthcare facilities"

Theme 1: Basic characteristics of women and interview information

1.1 Name of Region: __________________

1.2 Date_____/_____/_____

1.3 Interview code no. ______________

1.4 Interview date: ____________________

1.5 Interview start time: _______________

1.6 Interview end time: ________________

1.7 Location: ________________________

1.8 Marital status: ____________________

1.9 Ethnicity: ________________________

1.10 Religion: _______________________

1.14 Education: ______________________

1.15 Occupation: _____________________

1.16 Age: ____________

1.17 Total number of children: _________

1.18 Sex of child: ______

1.19 Age of child: ______

2.00 SES (perceived) _________________

Informed consent obtained __________________

Today we are going to talk about the health care need and utilization of women for the MNCH services. We will also discuss why some people utilize the MNCH services and others don't and the reasons in this regard in more detail. We will first talk about the common MNCH related problems and health care needs and utilization, and then about the disparity in the MNCH services utilization among the different socioeconomic statuses and some suggestions on how to narrow the disparity in the utilization of the services.

Theme 2: Common maternal, newborn and child health problems in the community

2.1 Now, I would like you to tell me about the common MNCH problems in your community. Tell me as much detail as you can remember.

Probe: for problems (during **pregnancy, delivery, and PNC)**: What type of problems (list all the problems mentioned)? How do they occur? How common they are? Why are these such a big problem in this community?

2.2 I would also like you to tell me about the common child health problems in your community. Tell me as much detail as you can remember.

Probe: What are the main child health-related problems in your community? How common they are? Why are these such big problems in this community?

Theme 3: Perception of maternal and child health care needs and utilization

3.1 Can we discuss the available maternal and child health services in your community. Tell me about the different maternal and child health services provided at the primary health care facilities.

3.2 Can you tell me about your healthcare need during pregnancy, delivery, and PNC?

Probe: 3.2.1 during pregnancy (where do you go to seek health care and why? when do you go there? who was involved in decision making?)

3.2.2 How about during delivery? (where do you go to seek health care and why? when do you go there? who was involved in decision making?)

3.2.3 How about during PNC? (where do you go to seek health care and why? when do you go there? who was involved in decision making?)

3.2.4 I would also like you to tell me about child health care? (where do you go to seek health care, when was the care-seeking for a sick child, who was involved in decision making?)

3.2.5 We have recruited you to this study because you have the experience of utilizing the health services for either of the services mentioned above. What triggers you to go to the health facility for the services?

3.3 What do you think people seek care other than the health facilities? (List all mentioned) What makes them do so?

3.3.1 Some people seek care at home. In your opinion, can you help us understand why this happens?

3.3.2 What other problems make mothers less likely to use the health facilities?

Probe: What are the other major problems deterring utilization?

Theme 4: Perceived difference in the MNCH services utilization

4.1 Tell me why some people use the services at the health facilities and others don't.

- What do you think the difference in the utilization of the services between poor versus wealthy, educated versus with no education, urban versus rural in your community? (During pregnancy, delivery, and PNC and child health services).

Probe: let's discuss one-by-one the utilization of the services in more detail.

4 How big are the differences?

5 Why do differences happen? What might explain some women utilize the services and others don't (cost, distance, transport, education, residence)? Anything else?

6 I would like to learn how do you feel about the differences in the utilization of the services.

7 Do you think the differences are fair?

4.2 What other barriers cause a difference in the MNCH services utilization at the primary health care facilities in your community?

4.3 In your opinion, do you think a difference in the utilization of the services affects the health of the mother or children? If yes, how and why?

Probe: - community beliefs?

- how about health care providers' behaviors? Do you think this affects health services utilization?

4.4 We are also interested to know about the HEWs' community outreach activities in this community on MNCH services.

Probe:

- What do they do when they move house-to-house? Let’s discuss this in more detail.
- How often do they come to your houses?
- Are there households frequently visited by the HEWs or spend more time with the households? If yes, in your opinion, who are these households (their socioeconomic status), and what do you think they are frequently visited by the HEWs?

Theme 5: Experiences of differences in the health services provision

5.1 We are interested to know if there are differential treatments for the maternal and child health services provided at the health facilities by the health care providers?

- Can you tell me about your experience when you visited the services? (for those who have ever visited the health facilities for any of the MNCH services mentioned above)

Probe:

- What service did you use it for?
- What happened at the facilities?
- How do you feel about using the services?

5.2 Do the health care providers differentiate during services provision by socioeconomic status or other factors?

- What do you think health care providers treat clients differently? And why? What are the reasons for differential service provision/treatment?

5.3 From your experience, did you hear that there is differential treatment at the facilities by type of services provided to clients?

5.4 Do you think unequal treatment by the health care providers affect the health of the mothers and children? If yes, how? What are the effects? How common is this problem at the health facilities?

5.5 What other major challenges did you face at the health facilities? Mention all.

Theme 6: Recommendations

6.1 Tell me if there are existing opportunities to narrow the disparity in service utilization in this community.

Probe: What other organizations are working in narrowing the disparity in the utilization of the services among the different population subgroups?

- Which organizations?
- what activities do they do?

- How do they accomplish their activities? And with whom do they perform the activities? Who is the target population for the organizations? Do they include people with low socioeconomic status and hard to reach areas?
- Did you find the activities are important in narrowing inequity in the utilization of the services? If so, do you further recommend the activities to implement in your community?

6.2 If the mother responds there is a difference in the utilization of the services among mothers with varied socioeconomic status in the community. Ask her what she thinks should be done at the health facility.
Probe:

- In your opinion, what do you think the healthcare providers can do to narrow the disparity in the utilization of the services?
- In your opinion, what do you think the health system can do to narrow the disparity in the utilization of the services?
- In your opinion, what do you think the community can do to narrow the disparity in services utilization?

Conclusion: Is there anything else that you want to tell me or think I should know about your experience of utilization of the MNCH services at the health facilities or the way you were treated at the health facilities?

Thank you very much for taking the time to participate in this interview.

Focus Group Discussion (FGD) guide for Women

Theme 1: Basic characteristics of participants

1.1 Name of Region: _________________

1.2 Date_____/_____/_____

1.3 FGD code no. _____________

1.4 FGD date: ___________________

1.5 FGD start time: ______________

1.6 FGD end time: _______________

1.7 Location: _______________________

1.8 Marital status of all participants: ___________________

1.9 Ethnicity: _______________________

1.10 Religion: ______________________

1.11 Education: _____________________

1.12 Occupation: ____________________

1.13 Age: ___________

1.14 Informed consent obtained _________________

Theme 2: Child health care practices

1. What are the common MNCH related problems in this community? List all diseases and problems mentioned.
2. What type of MNCH services provided at the health facilities? List all mentioned.
3. Where do mothers go when a need for either of the MNCH services (pregnancy, delivery, PNC, and care-seeking for child health)? Do they go to health facilities? If no, why?

Theme 3: MNC health services utilization

1. In this community, tell me the experience of mothers in utilizing the MNCH services.

Probe: In your opinion, how do you see the utilization of the services during pregnancy, delivery, PNC, and child health at the health facilities?

- For those women who utilize the services, what are the enabling factors that make women utilize the services? Let’s discuss the factors in more detail.
- For those women who don’t utilize the services, what are the factors that deter women not to utilize the services? Let’s discuss this in more detail.

Theme 4: Perceived difference in the MNCH services utilization

1. In this community, some women utilize MNCH services but others don’t. In your opinion, what do you think the reasons for the disparity in the utilization of the services?
   Probe: Do you think there is a disparity in the utilization of the services?
   - poor or rich? Why?
   - Educated or non-educated? Why?
2. What other factors do you think that causes a disparity in the MNCH services utilization?
   Probe: Do you think **cost**, husband's support, availability **of transport**, etc. can cause a disparity in the utilization of the services? Anything else?
3. Do you think the differences are fair?

Theme 5: Perception of the community about the provision of the services

1. Tell me about the service provision at the health facilities? How does the community perceive the services provided at the health facilities in terms of its fairness?
   - Do you think the services provided at the health facilities fair (have equal treatment for all women)? What is your opinion?
   - Are there differential treatments in the MNCH services? If yes, in your opinion, what do you think the HWs create disparity, and among whom?
2. How do you find the attitude of the HWs towards treating all population groups equally? Discuss in more detail.
3. What do you think the HWs treat clients differently? Detail more.
4. Tell me the experience of the HWs during outreach services. Where do they go most (especially the HEWs)?

   Probe: The poor or rich? Why? What else?

Theme 6: Recommendations

1. In your opinion, to minimize differences in the utilization of the services, what measures do you think should be done?

2. Are there community health workers working in improving the fairness of child health services utilization?

   Probe:

   - Who are they?
   - What do they do?
   - How is the perception of people towards these community health workers?

3. Are there other programs or projects implementing in improving equity in the child health services utilization at the health posts?

   Probe:

   - What is being done?
   - Which programs?
   - How is the perception of people towards these programs?

Key Informant Interviews (KIIs) with Health Workers at different positions

Theme 1: Basic characteristics of informants

1.1 Name of Region: ___________________

1.2 Date_____/_____/_____

1.3 Interview code no. ______________

1.4 Interview date: ____________________

1.5 Interview start time: _______________

1.6 Interview end time: ________________

1.7 Location: ________________________

1.8 Marital status: ____________________

1.9 Ethnicity: ________________________

1.10 Religion: _______________________

1.11 Education: ______________________

1.12 Occupation: _____________________

1.13 Profession ______________________

1.14 Age: ____________

1.15 Type of health facility _____________

Informed consent obtained __________________

Theme 2: MNCH services delivery practices

1. What MNCH services are provided at the health facilities? I would like to know how these services are being delivered at the health posts.

   Probe:

   - List all services mentioned.
   - Who is involved in the service delivery?
   - Could you explain to me how the HWs deliver the services to their clients?
   - Are they capable enough to deliver the services?
   - How is the implementation of the service monitored? What mechanisms do you use to monitor the services?

2. Who does the MNCH health services target at the health posts?

   Probe:

   - The poor or rich?
   - The educated or non-educated population subgroups? Who are the main utilizers of the service? Why?
   - Have you observed differences in the community in the utilization of the services? Who utilizes more?
   - Where do mothers go when in need of these services? Do they go to health facilities? If no, why?

Theme 3: MNCH services utilization

1. In this community, tell me the experience of mothers in utilizing the services at the health facilities in more detail.

   Probe:

   - What are the common MNCH problems? List all mentioned.
   - What are the community's perceptions and practices to these services' utilization?

Theme 4: Perceived difference in the MNCH services utilization

1. Some women utilize the services at the health facilities others do not. What do you think is the reason? Do you think there is a disparity in utilization by socio-economic status (wealth, education, cost, transport, etc)? Anything else, and how?
2. Do you think the differences are fair?

3. How do you describe the health policy for disadvantaged women who reside in the villages? Does the policy give any explicit indications to address the needs of those populations?
4. As a health worker, what is your role in narrowing the differences in service utilization among women in the different socioeconomic statuses? (**Probe**: What activities do you do so? How and when?
5. From your experience, when you talk to the community, what does the community say about the disparity in the utilization of the services? (**Probe**: Have you ever heard people are talking about the disparity? What did you hear? Please explain more.)

Theme 3: Perceived difference in the provision of the services

1. Tell me about the service provision at the health facilities by the HWs. How do you perceive the services provided in terms of fairness?

- Do you think the health services provided at the health facilities fair? What is your opinion?
- Is there a disparity in service provision by the HWs among women with varied socioeconomic status? If yes, why?
- How do you find the attitude of the HWs towards treating all population groups without disparity?

2. What do you think the HWs treat clients differently? Detail more.

Theme 4: Outreach services

1. Tell me the experience of the HWs including HEWs during outreach services. Are there households they frequently visit or spend more time with? Who are these households? And Why?

Theme 5: Recommendations

1. Are there other programs or projects working to improve fairness in the child health services provided to clients?

   Probe:

   - Which organizations?
   - What strategies do they implement?
   - What results did they achieve?

2. In your opinion, to increase the utilization of the services of the disadvantaged people, what do you think should be done (to the existing services or as a new strategy)? Explain.
3. In your opinion, to improve fairness in the provision of the services for sick children at the health posts, what measures do you think could be done? Explain.

Conclusion: Is there anything else that you want to tell me or think I should know about your experience of seeking the child health services at the health post or the way you were treated at the health post? *(Respond accordingly)*

Thank you very much for taking the time to participate in this interview.

Thank you and Goodbye.
